# Role of the Mouse Retinal Photoreceptor Ribbon Synapse in Visual Motion Processing for Optokinetic Responses

**DOI:** 10.1371/journal.pone.0124132

**Published:** 2015-05-08

**Authors:** Yuko Sugita, Fumiyuki Araki, Taro Chaya, Kenji Kawano, Takahisa Furukawa, Kenichiro Miura

**Affiliations:** 1 Department of Integrative Brain Science, Graduate School of Medicine, Kyoto University, Kyoto, Japan; 2 Laboratory for Molecular and Developmental Biology, Institute for Protein Research, Osaka University, Osaka, Japan; 3 CREST, Japan Science Technology Agency, Tokyo, Japan; 4 Department of Developmental Biology, Osaka Bioscience Institute, Osaka, Japan; 5 Department of Ophthalmology, University of Tokyo Graduate School of Medicine, Tokyo, Japan; Dalhousie University, CANADA

## Abstract

The ribbon synapse is a specialized synaptic structure in the retinal outer plexiform layer where visual signals are transmitted from photoreceptors to the bipolar and horizontal cells. This structure is considered important in high-efficiency signal transmission; however, its role in visual signal processing is unclear. In order to understand its role in visual processing, the present study utilized *Pikachurin*-null mutant mice that show improper formation of the photoreceptor ribbon synapse. We examined the initial and late phases of the optokinetic responses (OKRs). The initial phase was examined by measuring the open-loop eye velocity of the OKRs to sinusoidal grating patterns of various spatial frequencies moving at various temporal frequencies for 0.5 s. The mutant mice showed significant initial OKRs with a spatiotemporal frequency tuning (spatial frequency, 0.09 ± 0.01 cycles/°; temporal frequency, 1.87 ± 0.12 Hz) that was slightly different from the wild-type mice (spatial frequency, 0.11 ± 0.01 cycles/°; temporal frequency, 1.66 ± 0.12 Hz). The late phase of the OKRs was examined by measuring the slow phase eye velocity of the optokinetic nystagmus induced by the sinusoidal gratings of various spatiotemporal frequencies moving for 30 s. We found that the optimal spatial and temporal frequencies of the mutant mice (spatial frequency, 0.11 ± 0.02 cycles/°; temporal frequency, 0.81 ± 0.24 Hz) were both lower than those in the wild-type mice (spatial frequency, 0.15 ± 0.02 cycles/°; temporal frequency, 1.93 ± 0.62 Hz). These results suggest that the ribbon synapse modulates the spatiotemporal frequency tuning of visual processing along the ON pathway by which the late phase of OKRs is mediated.

## Introduction

The ribbon synapse is a specialized synaptic structure in the outer plexiform layer of the retina where visual signals are transmitted from photoreceptors to bipolar and horizontal cells [1, 2]. The structures of the ribbon synapse are thought to be important in high-efficiency signal transmission [1]. There are reports that an extracellular matrix protein, Pikachurin, is essential for the proper formation of the ribbon synaptic structure [3, 4]. *Pikachurin*-null mutant mice (*Pikachurin-/-*) do not have normal ribbon synaptic formation. In electrophysiological experiments, the *Pikachurin-/-* mice show reduced amplitude and prolonged implicit time of the scotopic and photopic electroretinogram (ERG) b-wave. These abnormalities indicate that the ribbon synaptic structures are important in visual function; however, their roles are not well understood.

Optokinetic responses (OKRs) are reflexive eye movements elicited by a moving visual pattern [5–13] and are powerful tools to discover visual properties [14–17]. Recently, the effect of retinal abnormalities on the mouse OKR has been studied [3, 18–20]. We previously studied the OKRs of the *TRPM1-/-* (transient receptor potential cation channel, subfamily M, number 1) and *mGluR6-/-* (metabotropic glutamate receptor 6) mice that have dysfunctional ON-bipolar cells, and found that the initial development of OKRs was mediated by both the ON and OFF pathways. In addition, the late phase of the OKRs observed as slow phase eye velocity of the optokinetic nystagmus (OKN), was mediated specifically by the ON pathway [20]. In the *Pikachurin-/-* mice, the optokinetic responses (OKRs) induced by rotation of a screen describing black and white stripes showed partial impairment of the OKR gain when the strip width was as narrow as 1.25 degrees, whereas no abnormalities were observed with wider strips [3]. There might be an influence of Pikachurin on spatiotemporal frequency characteristics in visual processing in the retina. However, because the previous study [3] used only a limited set of visual stimuli, the changes in visual characteristics remain unclear.

In the present study, to understand the role of the photoreceptor ribbon synapse in retinal visual motion processing, we examined the OKRs in *Pikachurin*-null mutant mice. We recorded the initial and late phases of OKRs of the *Pikachurin-/-* mice to sinusoidal gratings of various spatial frequencies drifting at various temporal frequencies. Our results revealed that the photoreceptor ribbon synapse contributes to the spatiotemporal frequency tuning of the retinal visual processing.

## Materials and Methods

Most of the techniques for the animal preparation, eye movement recording, and visual stimulation were similar to those described in previous studies [16, 17, 20]

### Animal preparation

Data were collected from 11 *Pikachurin-/-* mice and 13 wild-type 129Sv/Ev (Taconic) mice (control group), weighing 15.8–23.7 g (3–4-month-old). In order to minimize the number of animals used in the experiments, raw data of 10 of the 13 control mice were obtained from our database because the experimental procedures to acquire the data were the same. These raw data were used in our previous study [20]; however, the methods of analyses were different from those of the current study. Each mouse was previously implanted with a head holder, which allowed the head to be fixed in the stereotaxic position during the experiments. Mice were anesthetized with an i.p. injection of a mixture of ketamine hydrochloride and xylazine hydrochloride, and mounted on a stereotaxic apparatus (Narishige, Tokyo, Japan). After making an incision to expose the skull surface, the position was adjusted so that the bregma-lambda axis was horizontal and a head holder was fixed to the top of the skull using stainless steel screws and dental cement. The animal was allowed to fully recover after surgery before the first experiments were performed. During the experiments, the animal was restrained by bolting the attached head holder to a rigid rod at the center of the platform. We minimized the number of animals used and their suffering, and all experiments were performed in accordance with protocols approved by the Animal Care and Use Committee of Kyoto University and Osaka University and the guidelines laid down by the NIH in the US regarding the care and use of animals for experimental procedures.

### Eye movement recording

The right eye of the mouse was illuminated by infrared light-emitting diodes and monitored with a CCD camera. The eye-movement data were collected on a computer (Endeavor, Epson, Nagano, Japan) using image-processing software (Geteye, Matsuura-Denko-sha, Kanazawa, Japan). The software detected the center of the pupil and measured its position at intervals of 5 ms [16]. Recordings were performed in darkness to avoid contamination from irrelevant visually driven eye movements.

### Visual stimulation

The stimuli were presented on 19-inch computer monitors (spatial resolution, 1280 ×1024 pixels; refresh rate, 75 Hz; LCD, Mitsubishi, Tokyo, Japan). Three displays showing identical visual stimuli were set around the animal at the front and both sides, spanning 270°× 76.6°(azimuth × elevation) in the visual field. Each display was located at a distance of 19 cm from the center of the platform on which the head of the mouse was stereotaxically fixed. The eyes of the mouse were positioned 13 cm above the platform. The visual stimuli were drifting vertical sinusoidal gratings (Michelson contrast, 64%; mean luminance, 100 cd/m^2^) generated using MATLAB (Mathworks, Natick, MA, USA) and the Psychophysics Toolbox extensions [21]. We adjusted the spatial frequency of the grating on the computer monitors to mimic a rotating drum drifting at a constant speed as in our previous studies [16, 20].

### Procedures

In Experiment 1, we investigated the initial phase of the OKRs. The visual images were vertical sinusoidal gratings that could have one of five spatial frequencies selected randomly from a lookup table: 0.031, 0.063, 0.125, 0.25, and 0.5 cycles/° in a given trial. The speed of the visual stimulus was defined by the temporal frequency, which was selected from 0.1875, 0.375, 0.75, 1.5, 3, 6, 12, or 24 Hz. The duration of the visual stimulus motion was 500 ms. At least 10 trials were repeated for each stimulus condition and each mouse. The data on wild-type mice (*n* = 7), and *Pikachurin-/-* mice (*n* = 9) were thus obtained.

In Experiment 2, we examined the late (or sustained) phase of the OKRs induced by longer visual motion stimulations (30 s). The image patterns and speeds were the same as in Experiment 1. Trials were repeated for once or twice for each stimulus condition. The data on wild-type mice, (*n* = 10) and *Pikachurin-/-* mice, (*n* = 9) were obtained.

At the beginning of each trial in Experiment 1 and 2, a vertical sinusoidal grating pattern was presented on the monitors. The grating pattern was stationary for 333 ms and then moved either clockwise or counterclockwise. The speed of the visual stimulus was constant during each trial.

### Data analysis

All data were transferred to another PC for analysis using computer programs based on MATLAB (The MathWorks Inc.). The eye-position data were smoothed with a 4-pole digital Butterworth filter (-3 dB at 15 Hz), and then the eye velocity traces were derived from the two-point backward difference. Eye-acceleration profiles were also derived from the two-point backward difference of the eye velocity traces.

In Experiment 1, to examine the initial phase of the OKRs, the mean eye velocity during the 100-ms interval starting 100 ms after the onset of visual motion was calculated. Trials with saccadic intrusions (eye velocity > 30°/s, eye acceleration > 2000°/s^2^) during the 300-ms interval starting 100 ms before onset of the visual motion were discarded. To increase the signal-to-noise (S/N) ratio, the eye velocity in the direction of stimulus motion was averaged over counter-clockwise and clockwise motion for individual spatiotemporal frequencies and individual mice. The spatiotemporal frequency tuning of the responses was characterized using two-dimensional (2D) Gaussian functions of log-spatiotemporal frequency:

$$R\left(x,\;y\right)=\;r\text{ exp}\left(-\left(\;\sigma_{x}{}^{2}{\left(\;x-x_{0\;}\right)}^{2}+2\rho \sigma_{x}\sigma_{y}\left(\;x-x_{0\;}\right)\left(\;y-y_{0\;}\right)+\;\sigma_{y}{}^{2}{\left(\;y-y_{0\;}\right)}^{2}\right)\right)$$ (1)

where *x*_0_ and *y*_0_ denote the peak spatial and temporal frequencies and were optimized together with all the other free parameters, i.e., *r*, *σ*_*x*_, *σ*_*y*,_ and *ρ*. In the present study, the spatiotemporal frequency characteristics were examined for each mouse.

To estimate the latency of ocular responses, we applied receiver operating characteristic (ROC) analysis. We determined the time at which the eye velocity elicited by counterclockwise visual motion separated from that elicited by clockwise motion for each spatiotemporal frequency. We constructed the ROC curve every 5 ms and then calculated the areas under the ROC curves (AUC). The data were fitted to the Weibull distribution function:

$$y=q\times \left(q-0.5\right)\times \text{ exp}{\left(-t/r\right)}^{s}$$ (2)

where parameters *q*, *r*, and *s* were optimized. The time when the curve crossed 0.75 was determined as the latency of the ocular responses.

In Experiment 2, we analyzed the slow phase eye velocity of OKNs as the late phase of OKRs. The extraction of slow phases were carried out using the procedure described elsewhere [20]. The slow-phase eye velocity during the 20-s intervals starting 10 s after onset of the visual motion was averaged for each stimulus condition. When more than two trials were available for a stimulus condition, the average of these trials was calculated. We then evaluated the spatiotemporal tuning by the same procedure used in Experiment 1.

## Results

### Abnormalities in the initial OKRs

**Fig 1:**
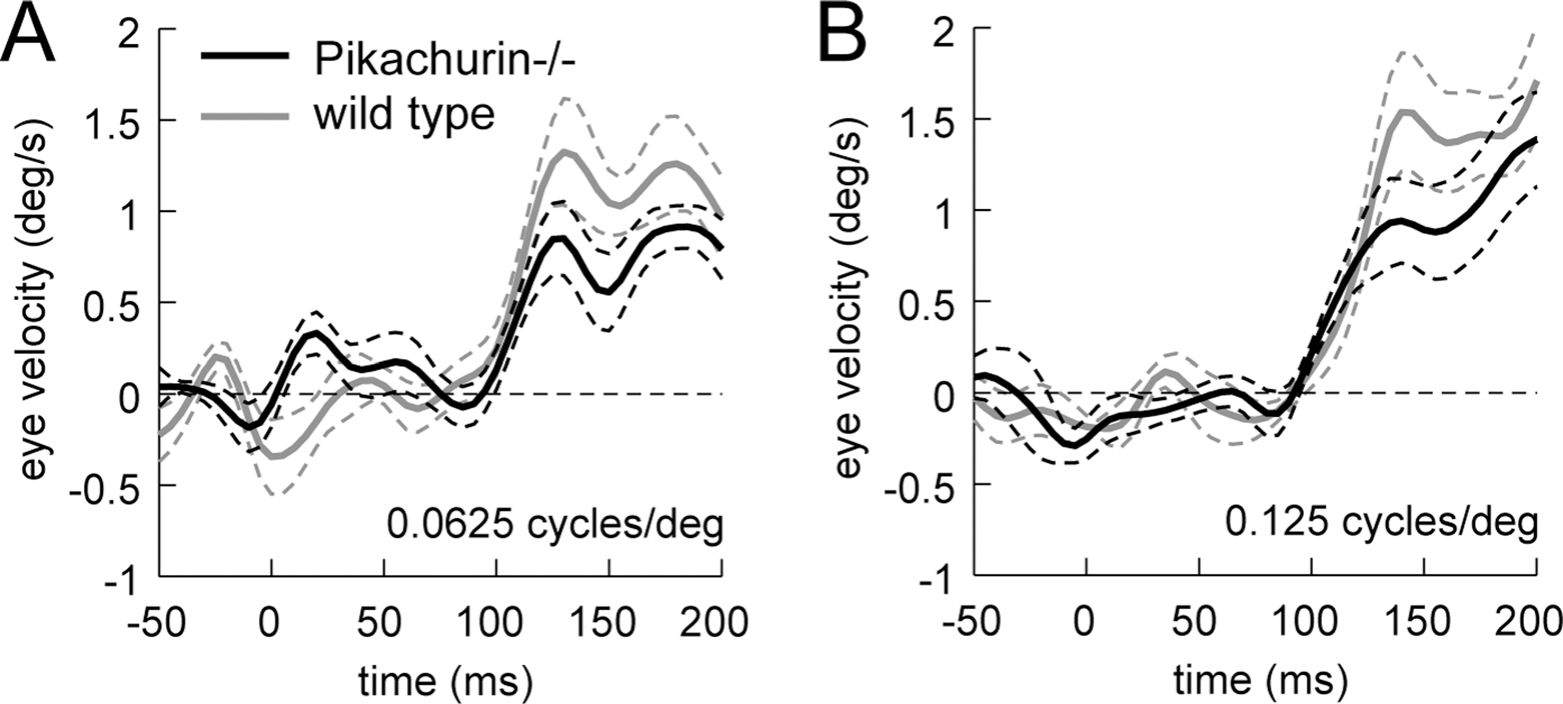
The optokinetic responses to motion of sinusoidal grating patterns drifting at temporal frequency of 1.5 Hz (A: spatial frequency, 0.0625 cycles/°; B: 0.125 cycles/°). Eye velocity profiles averaged over nine *Pikachurin-/-* mice (black solid lines) and seven wild-type mice (gray lines) are shown. The broken lines indicate standard error of the mean (SE) of the data on the *Pikachurin-/-* mice. ‘Time zero’ denotes the onset time of visual motion.

Fig 1 shows eye-velocity profiles averaged over the individual mice with the stimulus of spatial frequency of 0.0625 cycles/° and temporal frequency of 1.5 Hz (A) and with the stimulus of spatial frequency of 0.125 cycles/° and the same temporal frequency (1.5 Hz, B). The *Pikachurin-/-* mice showed clear optokinetic responses to moving sinusoidal gratings (Fig 1, AB, black lines) that were slightly smaller than those of the wild-type mice (gray lines). The response latencies were comparable with those of the wild-type mice as shown in Fig 1A and 1B. The statistical analysis of the latency measures obtained from ROC analyses showed no evidence of systematic latency change over the stimulus conditions (sign test, *p* = 0.18).

**Fig 2:**
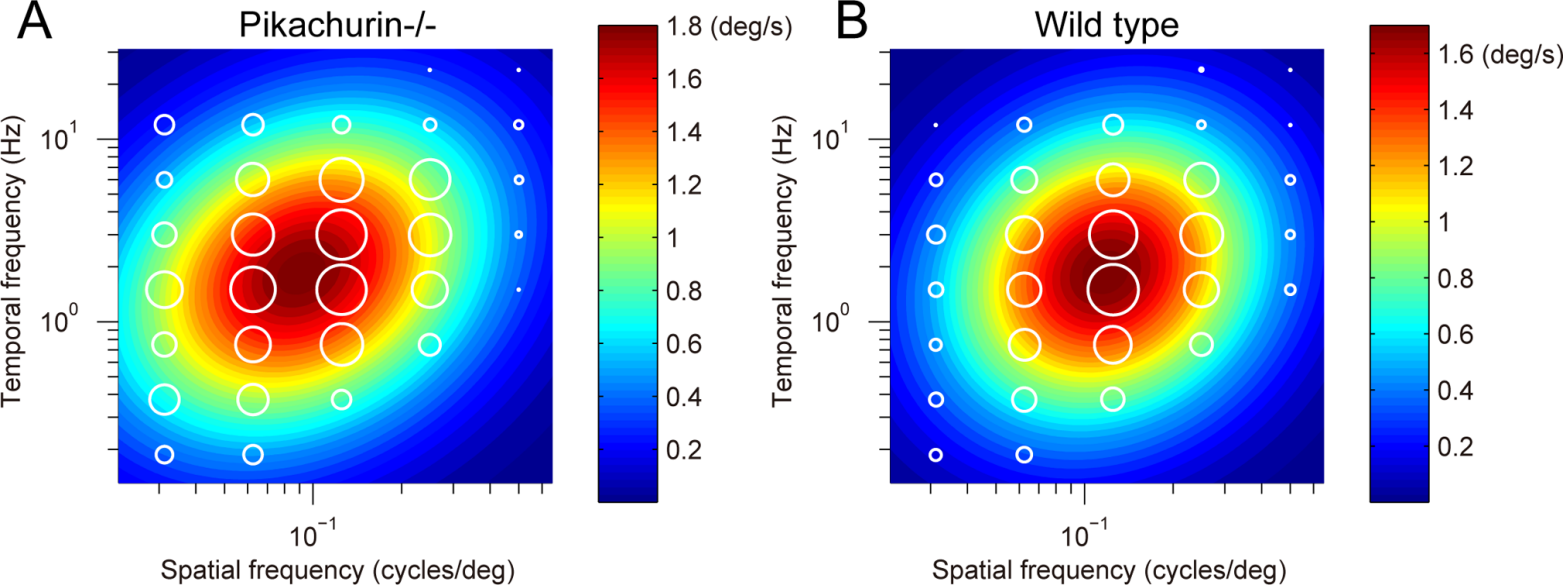
Dependence of initial OKRs on spatiotemporal frequency (A, a *Pikachurin-/-* mouse; B, a wild-type mouse). Amplitudes of the initial OKRs represented by diameter of the white circles are plotted in the coordinate system of spatial and temporal frequencies. Heat maps show the best-fit Gaussian functions.

The initial OKRs elicited by motion of sinusoidal gratings depended on spatiotemporal frequencies of the sinusoidal grating stimuli in both the *Pikachurin-/-* and the wild-type mice. An example of responses recorded in a *Pikachurin-/-* mouse is shown in Fig 2A. The visual responses were largest at a spatial frequency of 0.125 cycles/° and temporal frequency of 1.5 Hz in this mouse and the responses decreased as the spatiotemporal frequency deviated from this frequency. In order to estimate the optimal spatiotemporal frequency (*sf*_0_, *tf*_0_), a 2D Gaussian function of log spatiotemporal frequency was fitted to the data (*R* ^2^ = 0.83). The optimal spatial and temporal frequencies were estimated at 0.091 cycles/° and 1.80 Hz in this mouse. The data for the other mice could be characterized by the 2D Gaussian functions, and six of the nine *Pikachurin-/-* mice showed successful fit to the 2D Gaussian functions (*R* ^2^ > 0.56).

**Fig 3:**
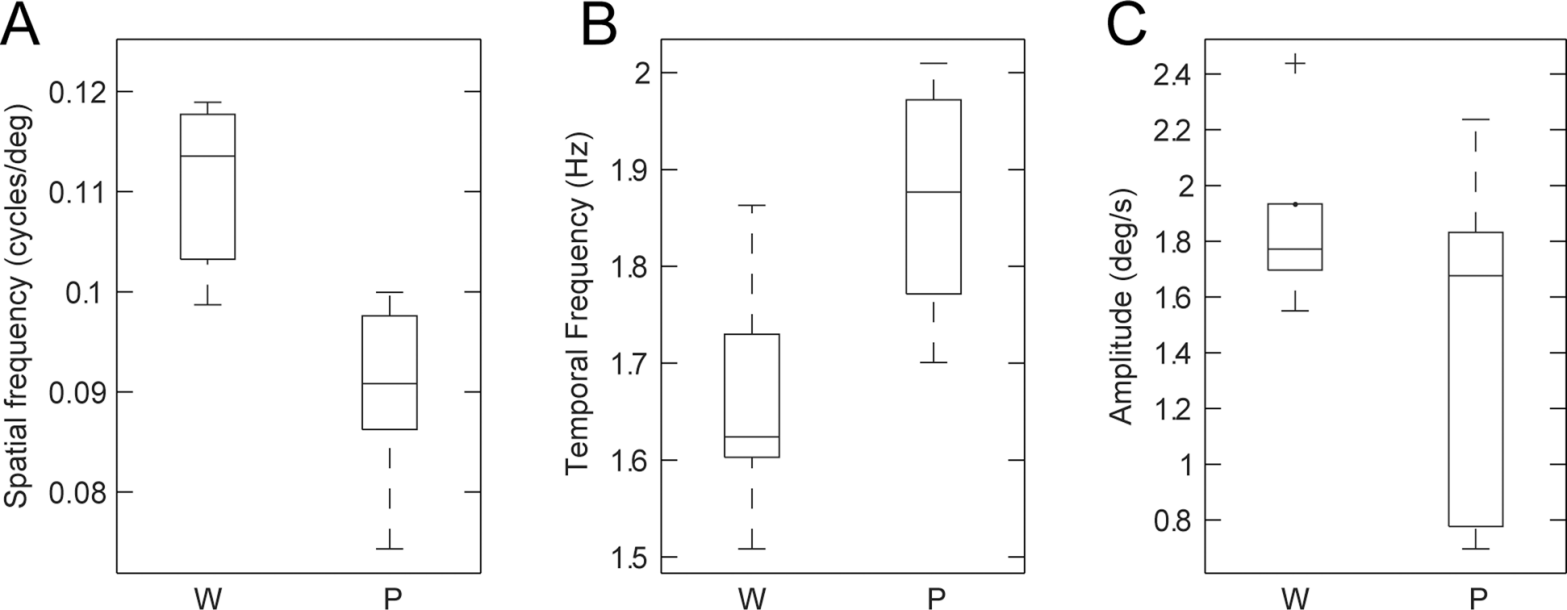
Comparisons of properties of the initial OKRs (six wild-type mice and six *Pikachurin-/-* mice). **A**: The difference in the optimal spatial frequency. **B**: The difference in the optimal temporal frequency. **C**: The peak amplitude of the response. W: wild-type mice, P: *Pikachurin-/-* mice.

The data for the wild-type mice could also be characterized by the 2D Gaussian functions, an example of which is shown in Fig 2B (optimal spatial frequency, 0.12 cycles/°; optimal temporal frequency, 1.9 Hz; *R* ^2^ = 0.92). Six of the seven wild-type mice showed successful fit to the 2D Gaussian functions (*R* ^2^ > 0.73). To evaluate whether these differences were significant, the optimal spatiotemporal frequencies were estimated from individual mice. The optimal spatial frequency was significantly different between the *Pikachurin-/-* mice (0.09 ± 0.01 cycles/°) and wild-type mice (0.11 ± 0.01 cycles/°) (Wilcoxon rank-sum test, *p* = 4.3 × 10^-3^) (Fig 3A). The difference in the optimal temporal frequency (*Pikachurin-/-* mice, 1.87 ± 0.12 Hz; wild type, 1.66 ± 0.12 Hz) was also significant (Wilcoxon rank-sum test, *p* = 2.6 × 10^-2^) (Fig 3B). The peak amplitude of the responses was not significantly different (*Pikachurin-/-* mice, 1.48 ± 0.62°/s; wild-type, 1.86 ± 0.31°/s) (Wilcoxon rank-sum test, *p* = 4.8 × 10^-1^) (Fig 3C). The spatiotemporal frequency tuning of the data averaged over all the mice (nine *Pikachurin-/-* and seven wild-type mice) showed similar differences in the optimal spatiotemporal frequency (spatial frequency: *Pikachurin-/-* mice, 0.09 cycles/°, wild type, 0.12 cycles/°; temporal frequency: *Pikachurin-/-* mice, 1.9 Hz, wild-type, 1.8 Hz; estimated with the best-fit 2D Gaussian functions).

### Abnormalities in the late OKRs

**Fig 4:**
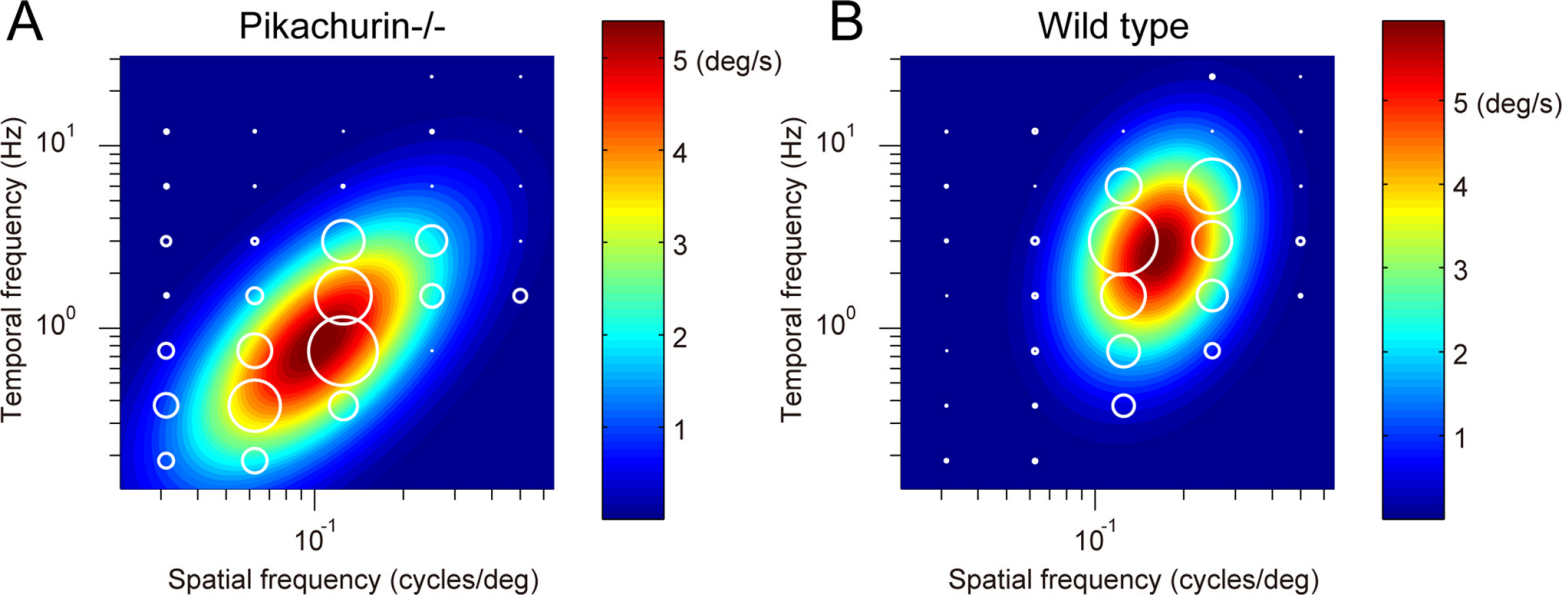
Dependence of the late phase of the OKRs on the spatiotemporal frequency. (A, a *Pikachurin-/-* mouse; B, a wild-type mouse). Mean amplitudes of the slow phase eye velocity of late OKRs represented by diameter of the white circles are plotted in the coordinate system of spatial and temporal frequencies. Heat maps show the best-fit Gaussian functions.

A prolonged exposure to the moving sinusoidal grating induced OKN that consisted of slow and quick movements in the *Pikachurin-/-* mice as well. The slow phase eye velocity during the OKNs as a measure of the late phase of the OKRs showed a clear spatiotemporal frequency tuning as well as the initial phase of the OKRs (Fig 4A). The optimal spatiotemporal frequency of a *Pikachurin-/-* mouse in Fig 4A estimated from the best-fit 2D Gaussian function (*R* ^2^ = 0.89) was 0.10 cycles/° and 0.82 Hz for spatial and temporal frequencies, respectively. Eight of the nine *Pikachurin-/-* mice showed successful fit to the 2D Gaussian functions (*R* ^2^ > 0.71).

**Fig 5:**
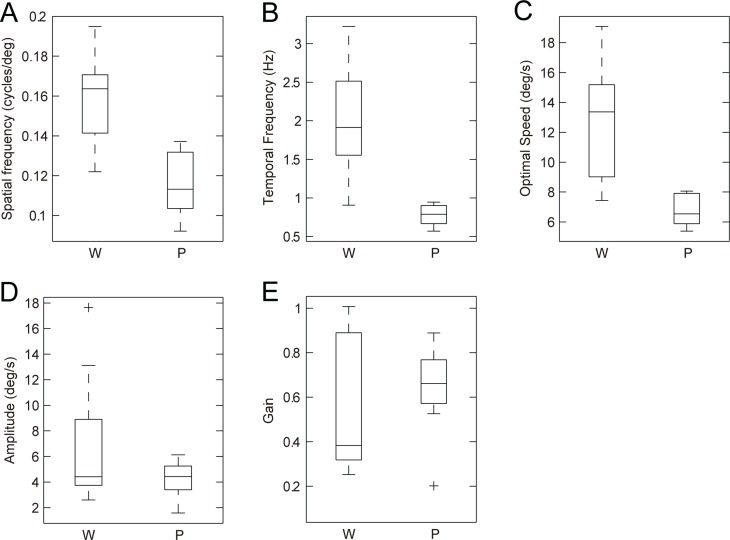
Comparisons of the properties of the late OKRs (seven wild-type mice and seven *Pikachurin-/-* mice). **A**: The difference in the optimal spatial frequency. **B**: The difference in the optimal temporal frequency. **C**: The stimulus speed at the optimal spatiotemporal frequency. **D**: The peak amplitude of the response. **E**: The gain at optimal stimuli. W: wild-type mice, P: *Pikachurin-/-* mice.

The data for the wild-type mice could also be characterized by the 2D Gaussian functions as shown in Fig 4B (one mouse, optimal spatial frequency, 0.16 cycles/°; optimal temporal frequency, 2.6 Hz; *R* ^2^ = 0.87). Nine of the ten wild-type mice showed successful fit to the 2D Gaussian functions (*R* ^2^ > 0.76). The optimal spatial frequency was significantly different between the *Pikachurin-/-* mice (0.12 ± 0.02 cycles/°) and wild-type mice (0.16 ± 0.02 cycles/°) (Wilcoxon rank-sum test, *p* = 2.5 × 10^-3^) (Fig 5A). The difference in the optimal temporal frequency (*Pikachurin-/-* mice, 0.78 ± 0.14 Hz; wild type, 2.04 ± 0.71 Hz) was also significant (Wilcoxon rank-sum test, *p* = 1.6×10^-4^) (Fig 5B). The speed of the optimal stimulus estimated by (optimal spatial frequency)/(optimal temporal frequency) was significantly different between the *Pikachurin-/-* (6.76±1.08°/s) and wild-type mice (12.79 ± 3.88°/s) (Wilcoxon rank-sum test, *p* = 5.8 × 10^-4^) (Fig 5C). The maximal attainable amplitude of the slow phase velocity was not significantly different (*Pikachurin-/-* mice, 4.24 ± 1.44°/s; wild-type, 7.01 ± 5.08°/s) (Wilcoxon rank-sum test, *p* = 4.8 × 10^-1^) (Fig 5D). Finally, the gain at the optimal stimulus was not significantly different (*Pikachurin-/-* mice, 0.64 ± 0.21; wild type, 0.54 ± 0.30; Wilcoxon rank-sum test, *p* = 5.4 × 10^-1^). The spatiotemporal frequency tuning of the data averaged across all the mice (nine *Pikachurin-/-* mice and 10 wild-type mice) showed similar differences in the optimal spatiotemporal frequencies (spatial frequency: *Pikachurin-/-* mice, 0.11 cycles/°, wild-type, 0.17 cycles/°; temporal frequency: *Pikachurin-/-* mice, 0.75 Hz, wild-type, 2.2 Hz; estimated with the best-fit 2D Gaussian functions).

The changes in OKR gain (eye velocity/stimulus velocity) were also dependent on spatiotemporal frequencies. The OKR gain of the *Pikachurin-/-* mice was generally smaller in higher spatiotemporal frequencies compared with the wild type mice with significant reduction at 0.25 cycles/° and 3.0 Hz. On the other hand, in lower spatiotemporal frequency, the OKR gain of the *Pikachurin-/-* mice was generally larger compared with that in the wild type mice with significant increases at four spatiotemporal frequencies (0.0625 cycles/° and 0.1875 Hz, 0.0625 cycles/° and 1.5 Hz, 0.125 cycles/° and 0.375 Hz, 0.125 cycles/° and 0.75 Hz). The OKR gain at the optimal spatiotemporal frequencies was not significantly different (Fig 5E). Taken together, the results imply that the absence of pikachurin protein in the null mutant mice did not simply result in reduced OKR performance, but resulted in different spatiotemporal frequency preferences compared to wild type mice.

## Discussion

In the present study, we examined detailed visual characteristics underlying OKRs of the *Pikachurin-/-* mice. No evidence for latency change was obtained. On the other hand, we observed abnormal spatiotemporal frequency characteristics on the initial as well as the late phases of the OKRs, with larger influence on the late phase.

### Use of *Pikachurin-/-* mice to study the function of ribbon synapses

The presynaptic interaction of Pikachurin with Dystroglycan at photoreceptor terminals is known to be essential for the formation of proper photoreceptor ribbon synaptic structures [4]. In the present study, we chose the Pikachurin^-/-^ mice to study the function of the photoreceptor ribbon synapses. The photoreceptor-specific Dystroglycan conditional knock out (CKO) mouse [4] might be an option to study the function of the photoreceptor ribbon synapses. However, Dystroglycan binds to various ligand proteins including Laminin, Perlecan, Agrin, and Pikachurin [3]. In addition, Omori et al. [4] showed that photoreceptor-specific Dystroglycan CKO mice showed more severe retinal phenotypes compared with *Pikachurin-/-* mice. Thus, Dystroglycan is likely to have additional functions in the retina [4]. Therefore, using *Pikachurin-/-* mice was scientifically more reasonable than using Dystroglycan CKO mice for the current purpose, i.e., to study the function of ribbon synapses.

### Comparisons with the previous studies

Sato et al [3] tested the slow phase of OKNs of the *Pikachurin-/-* mice induced by rotation of a screen of black and white stripes. They reported that the OKR gain was impaired when the stripe width was 1.25°, while responses were preserved when the stripe width was 15° or 1.92°. However, the reason for this impairment has not been identified because only four grating stimuli moving at a constant speed (10°/s) were used in their study; this impairment could be due to a change in spatial frequency tuning, a change in temporal frequency tuning, or both.

In the present study, we extensively investigated visual properties of the OKRs using systematic approaches and resolved the issue described above. First, the optimal temporal frequency in the late phase of the OKRs (slow-phase velocity in the OKN) of the *Pikachurin-/-* mice was much lower than that of the wild-type mice, which was the most remarkable difference. Second, the optimal spatial frequency tuning was lower in the *Pikachurin-/-* mice than in the wild-type mice. Thus, our present result suggests that the impaired OKR results from the change in both the spatial and temporal frequency tunings of the visual system underlying the OKR, although more weighted for the change in temporal frequency tuning. The impairment described by Sato et al. [3] is consistent with the inadequacy of the spatiotemporal frequency tuning in the present study. However, there is a quantitative difference in spatiotemporal frequencies at which the responses were impaired; this discrepancy might be explained by the differences in visual stimulations. Finally, we found that the optimal spatial frequency was lower also in the initial OKRs. Taken together, these results strongly suggest that the spatiotemporal characteristics in the retinal visual processing were affected by lack of Pikachurin expression.

We previously studied the OKR of the mutant mice (*TRPM1-/-* and *mGluR6-/-*) with dysfunction of the ON-bipolar cells, which have a functional obstruction of transmission to the ON-direction selective ganglion cells [20]. The mutant mice showed significant initial OKR with smaller amplitudes compared with the wild-type mice however, they did not show the late phase of the OKRs. The *Pikachurin-/-* mice showed significant initial and late OKRs, suggesting that the abnormalities in the OKRs were not due to destructive changes in the OKR pathways but due to relatively milder changes in the visual characteristics along the visual processing pathways in the retina.

### The role of Pikachurin in visual and oculomotor functions

The present study demonstrated that the optimal spatial frequency of OKRs was generally lower in the *Pikachurin-/-* mice compared with that in the wild-type mice. A difference in effective number of bipolar cells may explain this difference. In the *Pikachurin-/-* mice, fewer bipolar cells may tile the retina, resulting in coarse sampling of the visual space. Another possibility is a change in the receptive field (RF) structures of the bipolar cells involved in visual processing pathways in the retina. The center-surround antagonism is an important RF characteristic that determines the spatial frequency tuning characteristics with spatial extents of antagonistic center and surround regions. Center-surround antagonism has been found in the RFs of photoreceptor and bipolar cells, and the inhibitory signals from the horizontal cells contribute to form antagonistic surrounds [22–26]. In the *Pikachurin-/-* mice, the bipolar cell terminals are absent in the ribbon synapse, whereas the terminals of the horizontal cells are present [3]. Thus, the bipolar cells could lose interaction with the horizontal cells within the ribbon synapses. Thus, one possible mechanism underlying the change in the optimal spatial frequency is a reduced interaction between the horizontal and the bipolar cells. There have been no investigations on the frequency and the strength of the interaction. This point should be examined in future studies.

We also found that the optimal temporal frequency for the slow phase eye velocity of the OKN was lower in the *Pikachurin-/-* mice compared with that in the wild-type mice. This result suggests that the temporal characteristics for the retinal signal transmission are also affected in the *Pikachurin-/-* mice. As described above, the terminals of the bipolar cells are absent in the ribbon synaptic structure in the *Pikachurin-/-* mice. The different temporal characteristic might result from the structural difference in the ribbon synapse of the *Pikachurin-/-* mice than in wild type mice. In the normal ribbon synapse, photoreceptor cells provide signals to bipolar cells. The preference of lower temporal frequency in the *Pikachurin-/-* mice might be due to slower transmission from the photoreceptors to the bipolar cells.

Several lines of evidence suggest that the *Pikachurin* deletion specifically influences the ON pathway. The localization of mGluR6 is restricted to the postsynaptic site of ON bipolar cells in the ribbon synapses of the outer plexiform layer [27]. Almost of all of the pikachurin signals by immunostaining were found at the photoreceptor side of mGluR6, suggesting that Pikachurin localizes to the synaptic cleft of the ribbon synapse primarily around the postsynaptic terminals of ON bipolar cells but not of OFF bipolar cells [3]. Therefore, the OFF pathway is unlikely to be directly influenced by Pikachurin loss and disruption of ribbon synapses, although there is no direct evidence. Since OFF bipolar cells make flat contacts with photoreceptor cells [1], we suppose that Pikachurin is unnecessary for developing flat contact synapses.

The present results also suggest that the change in temporal characteristics occurs along the ON pathway which is known to mediate the late OKRs, i.e., slow-phase eye movements of the OKNs in mice [20]. This finding is in agreement with the findings from ERG examinations. *Pikachruin-/-* mice showed delayed and/or reduced ERG b-waves [3]. The b-wave originates in the retinal ON bipolar cells [28–30]. It should be noted that the reduced optimal temporal frequency was not observed in the initial OKRs, suggesting that this effect depends on the pathways for OKRs.

Pikachurin is expressed in the retina, lung, and ovary, but no significant expression has been identified in the brain [3]. Therefore, it is thought to have no direct effect on the central nervous system. On the other hand, we could not completely exclude an indirect effect on the central mechanism acquired through visual experiences. One way to test the effect on the central nervous system is to examine optokinetic after nystagmus (OKAN) which is a nystagmus observed after extinction of the optokinetic stimulus. The emergence of the OKAN is strongly species-dependent [8]. In the present study, we did not observe OKAN in mice. It has been suggested that mice do not show OKAN [31]. Therefore, another method must be developed to test this possibility.

### Retinal abnormalities and OKRs

Pikachurin is a ligand of Dystroglycan in the retina; and an interaction between Pikachurin and dystrophin-glycoprotein complex (DGC) is important for the normal formation of the ribbon synaptic structures [3]. In the present study, we demonstrated that the *Pikachurin-/-* mice showed abnormalities in spatiotemporal characteristics of the initial and late OKRs that could result from improper formations of the retinal ribbon synapses. This implies that similar eye movement abnormalities might also be observed in patients with muscular dystrophy having mutations of the DGC. If this is the case, the eye movement abnormalities can be used as a phenotypic output to test muscular dystrophy. However, there is no evidence showing a direct relationship between abnormalities in eye movement and mutations in the DGC. Future studies should be designed to clarify this point.
